# In-hospital outcomes after SAVR or TAVI in patients with severe aortic stenosis

**DOI:** 10.1007/s12928-023-00942-x

**Published:** 2023-06-22

**Authors:** Yasuaki Takeji, Tomohiko Taniguchi, Takeshi Morimoto, Shinichi Shirai, Takeshi Kitai, Hiroyuki Tabata, Kazuki Kitano, Nobuhisa Ohno, Ryosuke Murai, Kohei Osakada, Koichiro Murata, Masanao Nakai, Hiroshi Tsuneyoshi, Tomohisa Tada, Masashi Amano, Shin Watanabe, Hiroki Shiomi, Hirotoshi Watanabe, Yusuke Yoshikawa, Ryusuke Nishikawa, Ko Yamamoto, Yuki Obayashi, Mamoru Toyofuku, Shojiro Tatsushima, Norio Kanamori, Makoto Miyake, Hiroyuki Nakayama, Kazuya Nagao, Masayasu Izuhara, Kenji Nakatsuma, Moriaki Inoko, Takanari Fujita, Masahiro Kimura, Mitsuru Ishii, Shunsuke Usami, Kenichiro Sawada, Fumiko Nakazeki, Marie Okabayashi, Manabu Shirotani, Yasutaka Inuzuka, Kenji Ando, Tatsuhiko Komiya, Kenji Minatoya, on behalf of the CURRENT AS registry-2 Investigators, Takeshi Kimura

**Affiliations:** 1 Department of Cardiovascular Medicine, Graduate School of Medicine, Kyoto University, Kyoto, Japan; 2 Department of Cardiovascular Medicine, Kanazawa University Graduate School of Medical Sciences, Kanazawa, Japan; 3 Department of Cardiovascular Medicine, Kobe City Medical Center General Hospital, Kobe, Japan; 4 Department of Clinical Epidemiology, Hyogo College of Medicine, Nishinomiya, Japan; 5 Division of Cardiology, Kokura Memorial Hospital, Kitakyushu, Japan; 6 Department of Cardiovascular Medicine, National Cerebral and Cardiovascular Center, Suita, Japan; 7 Division of Cardiovascular Surgery, Kokura Memorial Hospital, Kitakyushu, Japan; 8 Department of Cardiology, Kurashiki Central Hospital, Kurashiki, Japan; 9 Department of Cardiology, Shizuoka City Shizuoka Hospital, Shizuoka, Japan; 10 Department of Cardiovascular Surgery, Shizuoka City Shizuoka Hospital, Shizuoka, Japan; 11 Department of Cardiovascular Surgery, Shizuoka General Hospital, Shizuoka, Japan; 12 Department of Cardiology, Shizuoka General Hospital, Shizuoka, Japan; 13 Department of Cardiology, Japanese Red Cross Wakayama Medical Center, Wakayama, Japan; 14 Division of Cardiology, Shimada General Medical Center, Shimada, Japan; 15 Department of Cardiology, Tenri Hospital, Tenri, Japan; 16 Department of Cardiology, Hyogo Prefectural Amagasaki General Medical Center, Amagasaki, Japan; 17 Department of Cardiovascular Center, Osaka Red Cross Hospital, Osaka, Japan; 18 Department of Cardiology, Kishiwada City Hospital, Kishiwada, Japan; 19 Department of Cardiology, Mitsubishi Kyoto Hospital, Kyoto, Japan; 20 Cardiovascular Center, The Tazuke Kofukai Medical Research Institute, Kitano Hospital, Osaka, Japan; 21 Department of Cardiology, Koto Memorial Hospital, Higashiomi, Japan; 22 Department of Cardiology, National Hospital Organization Kyoto Medical Center, Kyoto, Japan; 23 Department of Cardiology, Kansai Electric Power Hospital, Osaka, Japan; 24 Department of Cardiology, Japanese Red Cross Otsu Hospital, Otsu, Japan; 25 Division of Cardiology, Faculty of Medicine, Nara Hospital, Kinki University, Ikoma, Japan; 26 Department of Cardiology, Shiga General Hospital, Moriyama, Japan; 27 Department of Cardiovascular Surgery, Kurashiki Central Hospital, Kurashiki, Japan; 28 Department of Cardiovascular Surgery, Graduate School of Medicine, Kyoto University, Kyoto, Japan; 29 Department of Cardiology, Hirakata Kohsai Hospital, 1-2-1 Fujisaka Higashi-machi, Hirakata, 573-0153 Japan

**Keywords:** Aortic stenosis, Transcatheter aortic valve implantation, Surgical aortic valve replacement

## Abstract

There were no data comparing the in-hospital outcomes after transcatheter aortic valve implantation (TAVI) with those after surgical aortic valve replacement (SAVR) in Japan. Among consecutive patients with severe AS between April 2018 and December 2020 in the CURRENT AS Registry-2, we identified 1714 patients who underwent aortic valve replacement (TAVI group: 1134 patients, and SAVR group: 580 patients). Patients in the TAVI group were much older (84.4 versus 73.6 years, *P* < 0.001) and more often had comorbidities than those in the SAVR group. In-hospital death rate was numerically lower in the TAVI group than in the SAVR group (0.6% versus 2.2%). After excluding patients with dialysis, in-hospital death rate was very low and comparable in the TAVI and SAVR groups (0.6% versus 0.8%). The rates of major bleeding and new-onset atrial fibrillation during index hospitalization were higher after SAVR than after TAVI (72% versus 20%, and 26% versus 4.6%, respectively), while the rate of pacemaker implantation was higher after TAVI than after SAVR (8.1% versus 2.4%). Regarding the echocardiographic data at discharge, the prevalence of patient-prosthesis mismatch was lower in the TAVI group than in the SAVR group (moderate: 9.0% versus 26%, and severe: 2.6% versus 4.8%). In this real-world data in Japan, TAVI compared with SAVR was chosen in much older patients with more comorbidities with severe AS. In-hospital death rate was numerically lower in the TAVI group than in the SAVR group.

**Supplementary Information:**

The online version contains supplementary material available at 10.1007/s12928-023-00942-x.

Aortic valve replacement (AVR) is the only way to improve clinical outcomes in patients with severe aortic stenosis (AS). In the current era, transcatheter aortic valve implantation (TAVI) and surgical aortic valve replacement (SAVR) are the interventional options for patients with severe AS and have been reported to improve clinical outcomes compared to conservative therapy [1]. Several randomized controlled trials (RCTs) demonstrated comparable outcomes between SAVR and TAVI in patients with high to low surgical risk [2–6]. Based on these favorable outcomes of patients who underwent TAVI, the number of TAVI procedures has increased worldwide and the difference in age between SAVR and TAVI patients has decreased [7, 8]. However, there is a scarcity of data evaluating the practice pattern in the selection of treatment strategies for severe AS (TAVI, SAVR, or conservative), and comparing in-hospital outcomes between TAVI and SAVR in contemporary daily clinical practice [9]. Therefore, the aim of this study was to evaluate the patient characteristics and in-hospital outcomes in patients who underwent TAVI or SAVR in a large real-world Japanese registry of consecutive patients with severe AS in the current TAVI era.

## Methods

### Study population

Contemporary outcomes after sURgery and medical tREatmeNT in patients with severe Aortic Stenosis (CURRENT AS) registry-2 is a prospective, physician-initiated, non-company sponsored, multi-center registry enrolling consecutive patients who were diagnosed as having severe AS between April 2018 and December 2020 among 21 participating centers in Japan (Supplemental Appendix A). There were 10 centers where both SAVR and TAVI were available, 10 centers where only SAVR was available, and 1 center where neither SAVR nor TAVI was available. Inclusion and exclusion criteria of the CURRENT AS Registry-2 were described previously and were identical to those in the CURRENT AS Registry-1 [10, 11]. We defined patients with severe AS in this study as those who met at least one of the 3 echocardiographic criteria (peak aortic jet velocity [V_max_] > 4.0 m/s, mean aortic pressure gradient [PG] > 40 mmHg, or aortic valve area [AVA] < 1.0 cm^2^) for the first time during the enrollment period.

**Fig. 1:**
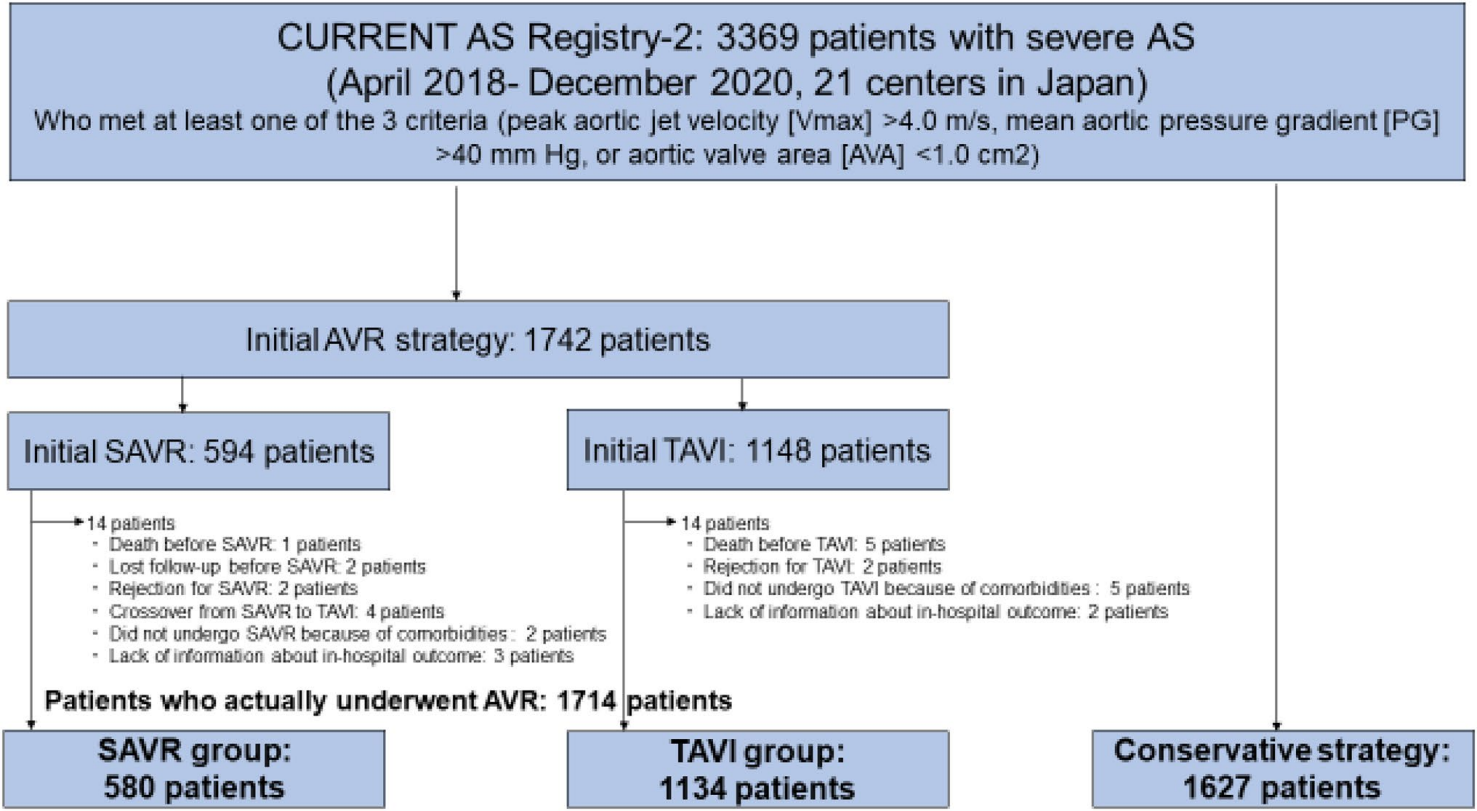
Study flowchart

Initial treatment strategies (initial AVR or conservative) were determined by the discussion between the attending physician and the patients with occasional heart-team consultation. The choice of initial SAVR strategy or initial TAVI strategy was made based on the consensus in the heart team and the preference of the patients. Among 3369 patients enrolled in the CURRENT AS Registry-2, the current study population consisted of 1742 patients (51.7%) in whom initial AVR strategy was selected (initial TAVI strategy: 1148 patients [34.0%], and initial SAVR strategy: 594 patients [17.6%]) after excluding 1628 patients (48.3%) in whom conservative strategy was selected. Finally, 1714 patients actually underwent AVR (TAVI group: 1134 patients [66.1%], and SAVR group: 580 patients [33.9%]) (Fig. 1). The Number of cases in each participating center was described in Supplemental table 1.

The follow-up was commenced on the day of AVR. The protocol was approved by the institutional review boards in all 21 participating centers. Written informed consent was obtained from each patient in 19 centers, while in the remaining 2 centers, the opt-out strategy waiving written informed consent was adopted with permission by the institutional review boards.

### Data collection and clinical outcomes

Details of data collection and definitions were described previously [11]. For the current analysis, the primary outcome measure was in-hospital death. The secondary outcome measures were stroke, disabling stroke, major bleeding, newly diagnosed atrial fibrillation (AF), pacemaker implantation, acute kidney injury, major vascular complication, intensive care unit stay after the procedure, hospital stay after the procedure, and hemodynamic parameters by echocardiography. In this study, we only evaluated those events, which occurred during or after AVR and up to hospital discharge. The definitions of clinical outcomes were described in Supplemental Appendix B. Clinical event committee adjudicated death, symptomatic stroke, and major bleeding (Supplemental Appendix C).

### Statistical analysis

We compared the patient characteristics, echocardiographic characteristics, procedural parameters, and in-hospital outcomes between the SAVR and TAVI groups. Moreover, patients’ characteristics were evaluated according to the 3 age categories (< 75 years, ≥ 75 years and < 80 years, and ≥ 80 years). Regarding clinical outcomes, we conducted a sub-group analysis stratified by the presence of dialysis, because TAVI for patients with dialysis was not approved in Japan during the enrollment period of the present study. We also conducted a sub-group analysis stratified by the presence of concomitant procedure, bicuspid aortic valve (BAV), coronary artery disease (CAD), any valvular disease, and STS score categories.

We presented categorical variables as numbers and percentages and continuous variables as mean ± standard deviation or median with interquartile range (IQR). We compared patient characteristics using the *χ*^2^ test for categorical variables and Student’s *t*-test or Wilcoxon rank-sum test for continuous variables based on their distributions. Because the selection of SAVR and TAVI was based on not only the patient characterisitics, but also other factors, the selection bias was inevitable. Therefore, we reported the in-hospital events only numerically as number and percentages without hypothesis testing. In addition, we did not construct the multivariable models because of the small number of events for the large differences in many patient characteristics. A two-sided *P* value < 0.05 was regarded as statistically significant for all tests. All analyses were performed using R version 3.6.1 (R Foundation for Statistical Computing, Vienna, Austria).

## Results

### Baseline characteristics

**Fig. 2:**
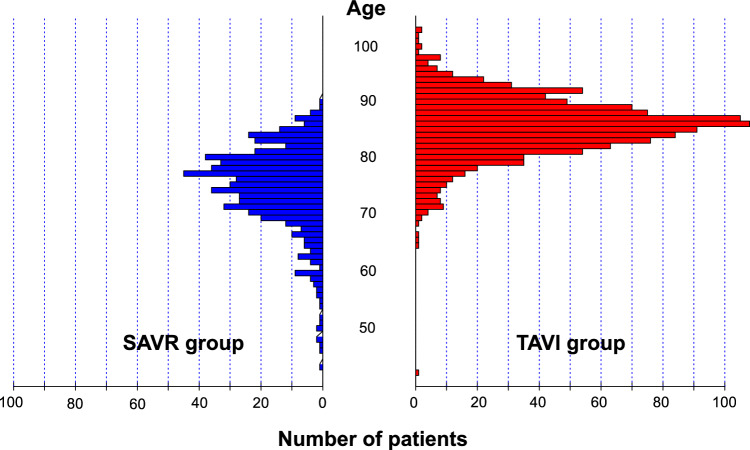
Distribution of Age in the SAVR and TAVI groups

Patients in the TAVI group were much older than those in the SAVR group (mean age: 84.4 years versus 73.6 years, P < 0.001) (Table 1, Supplemental Table 2). Patients with ≥ 80 years of age were predominantly treated with TAVI, while patients with < 80 years of age were more often treated by SAVR (Fig. 2). In the SAVR group, 52% of patients were men, while in the TAVI group, only 35% of patients were men. Society of Thoracic Surgeons-Predicted Risk Of Mortality (STS-PROM) score was higher in the TAVI group than in the SAVR group (4.6% versus 2.9%, P < 0.001). Patients in the TAVI group more often had comorbidities such as prior coronary artery bypass grafting (CABG), end-stage renal disease not on dialysis, anemia, moderate to severe chronic lung disease, and malignancy currently under treatment than patients in the SAVR group. Clinical frailty scale was much higher in the TAVI group than in the SAVR group. Nevertheless, patients with dialysis were almost exclusively treated by SAVR (Table 1, Supplemental table 2).

**Table 1:** Patient characteristics

	SAVR group	TAVI group	*P* value
	(*N* = 580)	(*N* = 1134)	
(A) Clinical characteristics			
Age (years)	73.6 ± 7.6	84.4 ± 5.6	< 0.001
Age ≥ 80 years	115 (20%)	962 (85%)	< 0.001
Men	304 (52%)	396 (35%)	< 0.001
Hypertension	436 (75%)	956 (84%)	< 0.001
Current smoking	42 (7.2%)	31 (2.7%)	< 0.001
Dyslipidemia	310 (53%)	588 (52%)	0.53
Diabetes mellitus	177 (31%)	303 (27%)	0.10
on insulin therapy	28 (4.8%)	40 (3.5%)	0.19
Prior myocardial infarction	24 (4.1%)	70 (6.2%)	0.08
Prior PCI	63 (11%)	156 (14%)	0.09
Prior CABG	4 (0.7%)	36 (3.2%)	0.001
Prior open heart surgery	14 (2.4%)	49 (4.3%)	0.047
Prior symptomatic stroke	53 (9.1%)	158 (14%)	0.004
Atrial fibrillation or flutter	104 (18%)	246 (22%)	0.07
Aortic and/or peripheral vascular disease	44 (7.6%)	68 (6.0%)	0.21
Dialysis	105 (18%)	2 (0.2%)	< 0.001
Anemia	233 (40%)	719 (63%)	< 0.001
Malignancy	98 (17%)	230 (20%)	0.09
Malignancy currently under treatment	22 (3.8%)	69 (6.1%)	0.045
Chronic lung disease	180 (31%)	381 (34%)	0.28
Chronic lung disease (moderate or severe)	22 (3.8%)	106 (9.3%)	< 0.001
Coronary artery disease	226 (39%)	448 (40%)	0.83
Clinical Frailty Scale			< 0.001
1–3	423 (73%)	550 (49%)	
4–6	151 (26%)	531 (47%)	
7–9	6 (1.0%)	53 (4.7%)	
STS PROM, %	2.9 (1.8–4.5)	4.6 (3.3–6.3)	< 0.001
(B) Presentation			
Etiology			< 0.001
Degenerative	440 (76%)	1093 (96%)	
Congenital (unicuspid, bicuspid, or quadricuspid)	125 (22%)	34 (3.0%)	
Rheumatic	11 (1.9%)	7 (0.6%)	
Infective endocarditis	3 (0.5%)	0 (0.0%)	
Other	1 (0.2%)	0 (0.0%)	
Symptoms			
AS related symptoms	441 (76%)	946 (83%)	< 0.001
Chest pain	95 (22%)	129 (14%)	< 0.001
Syncope	40 (9.1%)	114 (12%)	0.10
Heart failure	362 (82%)	837 (89%)	0.001
NYHA class			0.052
II	256 (71%)	532 (64%)	
III	81 (22%)	239 (29%)	
IV	25 (6.9%)	66 (7.9%)	

Anemia was defined as serum hemoglobin < 12 g/dl for women or < 13 g/dl for men, Coronary artery disease was defined to be present when meeting at least one of the following criteria: a history of PCI or CABG, a history of myocardial infarction, presentation as myocardial infarction at baseline, or angiographically confirmed coronary artery disease at baseline

*AVR* aortic valve replacement, *TAVI*  transcatheter aortic valve implantation, *SAVR*  surgical aortic valve replacement, *PCI * percutaneous coronary intervention, *CABG*  coronary artery bypass grafting, *STS*  society of thoracic surgeons, *PROM*  predicted risk of mortality, *NYHA* New York Heart Association

**Table 2:** In-hospital outcomes

	SAVR group	TAVI group	95% CI
	(*N* = 580)	(*N* = 1134)	
All-cause death	13 (2.2%)	7 (0.6%)	1.6 (0.3–2.9)
Stroke	12 (2.1%)	32 (2.8%)	− 0.8 (− 2.3–0.8)
Disabling stroke	5 (0.9%)	21 (1.9%)	− 1.0 (− 2.1–0.1)
Major Bleeding	416 (72%)	222 (20%)	52.1 (47.8–56.5)
Newly diagnosed AF	153 (26%)	52 (4.6%)	21.8 (18–25.6)
Pacemaker implantation	14 (2.4%)	92 (8.1%) SAPIEN3 47 (6.0%) Evolut R/ Pro/Pro Plus 45 (13.1%)	− 5.7 (− 7.7–3.7)
Acute kidney injury	32 (5.5%)	34 (3.0%)	2.5 (0.4–4.6)
Major vascular complications	20 (3.4%)	26 (2.3%)	1.2 (− 0.6–2.9)
ICU stay after procedure (days)	3 (2–4)	1 (1–2)	–
Hospital stay after procedure (days)	16 (13–23)	8 (7–12)	–

The definitions of the outcome measures were described in the Supplemental Appendix B

*ICU*  intensive care unit, *AF*  atrial fibrillation

The etiology of AS was degenerative in 96% of patients in the TAVI group, while it was congenital in 22% of patients in the SAVR group. More patients in the TAVI group had symptoms probably related to AS than those in the SAVR group.

Regarding the echocardiographic variables, severity of AS and flow pattern were not different between the SAVR and TAVI groups. Any combined valvular disease was more prevalent in the SAVR group than in the TAVI group (28% versus 23%, *P* = 0.02). Nevertheless, the prevalence of moderate or severe mitral regurgitation was comparable between the 2 groups (11% versus 11%, *P* = 0.78) (Supplemental Table 3).

**Table 3:** Echocardiographic data at discharge

	SAVR group	TAVI group	*P* value	N of patients evaluated
	*N* = 564	*N* = 1100		*N* = 1664
Vmax (m/s)	2.32 ± 0.44	2.24 ± 0.45	0.001	1653
Mean aortic PG (mm Hg)	11.9 ± 4.9	11.0 ± 4.8	0.001	1647
EOA (cm^2^)	1.57 ± 0.39	1.79 ± 0.48	< 0.001	1365
LVEF, %	58.6 ± 10.9	61.2 ± 9.9	< 0.001	1662
LV end-diastolic diameter (mm)	44 ± 6	44 ± 6	0.02	1662
LV end-systolic diameter (mm)	30 ± 7	29 ± 6	< 0.001	1641
LV Mass	167 ± 53	165 ± 46	0.44	1642
LVMI (g/m^2^)	103 ± 30	110 ± 28	< 0.001	1642
Stroke volume (ml/m^2^)	62 ± 17	72 ± 20	< 0.001	1294
Paravalvular aortic regurgitation ≥ moderate	0 (0%)	0 (0%)	–	1682
Patient–prosthesis mismatch^*^			< 0.001	1365
Moderate	85 (26%)	93 (9.0%)		
Severe	16 (4.8%)	27 (2.6%)		

EOA was measured by using equation of continuity

^*^Moderate Patient-Prosthesis Mismatch was defined as indexed effective orifice area (cm^2^/m^2^) 0.85–0.65 cm^2^/m^2^ for BMI < 30 kg/m^2^, OR 0.70–0.55 cm^2^/m^2^ for BMI > 30 kg/m^2^. Severe Patient-Prosthesis Mismatch was defined as indexed effective orifice area (cm^2^/m^2^) < 0.65 cm^2^/m^2^ for BMI < 30 kg/m^2^, OR < 0.55 cm^2^/m^2^ for BMI > 30 kg/m^2^

*V*_*max*_ peak aortic jet velocity, *PG* pressure gradient, *EOA* effective orifice area; *AVA* aortic valve area, *AS* aortic stenosis, *LV* left ventricular, *LVEF* left ventricular ejection fraction, *LVMI* left ventricular mass index, *BMI* body mass index

In terms of patient characteristics categorized by age, among patients younger than 75 years, those in the TAVI group were older, frailer, and more frequently presented with comorbidities such as prior CABG, symptomatic stroke, anemia, malignancy, immunosuppressive therapy, and moderate or severe chronic lung disease compared to patients in the SAVR group (Supplemental table 4).

Regarding patient characteristics in the SAVR group dividing with/without dialysis, patients with dialysis exhibited a higher prevalence of comorbidities including prior symptomatic stroke, atrial fibrillation or flutter, anemia, aortic and/or peripheral vascular disease, and coronary artery disease. Furthermore, patients with dialysis were more frail and had higher surgical risk scores compared to those without dialysis (Supplemental table 5).

### Procedural characteristics

In the SAVR group, 48% of patients underwent concomitant procedures such as CABG (28%), mitral valve surgery (9.5%), replacement of ascending aorta (8.4%), Maze procedure (7.4%), and tricuspid valve surgery (5.5%). Bioprosthetic valve was used in 96% of patients. The most frequently used bioprosthetic valve size was 21 mm followed by 23 mm valve (Supplemental table 6).

In the TAVI group, PCI before TAVI was conducted in a separate session from TAVI in 6.0% of patients and in the same session with TAVI in 0.9% of patients. TAVI procedure was performed under local anesthesia with conscious sedation in 61% of patients, and through transfemoral approach in 92% of patients. Balloon-expandable valve (SAPIEN 3; Edwards Lifesciences, CA, USA) was used in 69% of patients. The most frequently used valve size was 23 mm followed by 26 mm valve for the balloon-expandable valve, and 26 mm followed by 29 mm valve for the self-expandable valve (Supplemental table 6).

### In-hospital outcomes

Intensive care unit stay after the procedure was shorter in the TAVI group than in the SAVR group (1 [1–2] days versus 3 [2–4] days) and hospital stay after the procedure was also shorter in the TAVI group than in the SAVR group (8 [7–12] days versus 16 [13–23] days) (Table 2).

In-hospital death rate was numerically lower in the TAVI group than in the SAVR group (0.6% versus 2.2%). The rate of stroke was similar in the TAVI and SAVR groups (2.8% versus 2.1%). The rates of major bleeding and newly diagnosed AF were much lower in the TAVI group than in the SAVR group (20% versus 72%, and 4.6% versus 26%, respectively). In contrast, the rate of pacemaker implantation was higher in the TAVI group than in the SAVR group (8.1% versus 2.4%) (Table 2). The rate of pacemaker implantation in the TAVI group was higher with self-expandable valve than with balloon-expandable valve (13.1% versus 6.0%). The rate of acute kidney injury was numerically lower in the TAVI group than in the SAVR group (3.0% versus 5.5%).

Among patients without dialysis, in-hospital death rate was very low and comparable in the TAVI and SAVR groups (0.6% versus 0.8%) (Supplemental table 7). In contrast, among 105 patients with dialysis in the SAVR group, in-hospital death rate was high (8.6%), and the rates of disabling stroke and major vascular complications were much higher than those in patients without dialysis (Supplemental table 7 and 8). Among patients who underwent SAVR, in-hospital death rate of patients without concomitant procedure was numerically very low and lower than those with concomitant procedure (0.3% versus 4.3%) (Supplemental table 9). Among patients who underwent AVR for BAV, in-hospital death and disabling stroke rates were numerically higher in the TAVI group than in the SAVR group (2.9% versus 0.8%, and 5.9% versus 0.0%) (Supplemental table 10). With respect to patients stratified based on the STS-score risk stratum, the in-hospital mortality rate was remarkably high in SAVR group with high risk. In contrast, the in-hospital mortality rate in TAVI group was favorable even among patients deemed high risk. Moreover, even among low-risk patients, the in-hospital mortality rate was numerically lower in the TAVI group (Supplemental table 11). Among patients with CAD or any valvular disease, in-hospital death was numerically much higher in the SAVR group than in the TAVI group (4.4% versus 0.4%, 5.6% versus 0.4%) (Supplemental table 12, 13).

### Echocardiographic data at discharge

Echocardiographic data at discharge was available in 564 patients (97.2%) in the SAVR group and in 1100 patients (97.1%) in the TAVI group. Vmax and mean PG were higher in the SAVR group than in the TAVI group and EOA was larger in the TAVI group than in the SAVR group. Left ventricular ejection fraction (LVEF) and stroke volume were greater in the TAVI group than in the SAVR group. Patient-prosthesis mismatch was more often seen in the SAVR group than in the TAVI group (moderate: 26% versus 9.0%, and severe: 4.8% versus 2.6%) (Table 3).

## Discussion

The main findings of this study were the following: 1) Overall, among 3369 patients with severe AS enrolled from 21 centers, initial TAVI and initial SAVR strategies were chosen in 34.0% and 17.6% of patients, respectively; 2) Patients in the TAVI group were much older and had higher surgical risk scores than patients in the SAVR group, while patients with dialysis were almost exclusively treated with SAVR; 3) In-hospital death rate was numerically lower in the TAVI group than in the SAVR group. Among patients without dialysis, In-hospital death rate in patients was very low and comparable in the TAVI and SAVR groups (0.6% versus 0.8%), while in-hospital death rate in patients with dialysis was high after SAVR (8.6%); 4) In-hospital death rate of patients without concomitant procedure was numerically very low in the SAVR group (0.3%); 5) Among patients with BAV who underwent AVR, in-hospital death and stroke rates were numerically higher in the TAVI group than in the SAVR group (2.9% versus 0.8%, and 5.9% versus 0.0%); 6) Regarding the echocardiographic data at discharge, EOA, LVEF, and stroke volume were greater in the TAVI group than in the SAVR group, and the prevalence of patient-prosthesis mismatch was higher in the SAVR group than in the TAVI group.

The prevalence of initial AVR strategy in the present study (51.7%) was much higher than that in the CURRENT AS-1 registry (31.4%) conducted in the pre-TAVI era [10]. Moreover, the number of patients who underwent TAVI was much higher than the number of patients who underwent SAVR in this study. Suboptimal prevalence of SAVR had been one of an important issues in the management of patients with symptomatic severe AS [12]. Introduction of TAVI undoubtedly increased the prevalence of AVR particularly in patients with high or prohibitive surgical risk.

Currently, TAVI has been selected even in younger patients in the US after the low-risk trials [7]. In contrast, there was still a large difference in age between the TAVI and SAVR groups in the present Japanese study, and patients who underwent TAVI in this study were older than those in the US national registry [7]. Nevertheless, the in-hospital death rate after TAVI was numerically lower than that after SAVR. However, when considering the very low incidence of in-hospital mortality in patients without dialysis and patients without a concomitant procedure, the higher incidence of in-hospital mortality in the SAVR group was largely attributed to patients with dialysis or concomitant procedures.was very low and comparable to that after SAVR despite higher risk profiles of patients in the TAVI group than in the SAVR group. The in-hospital death rate after SAVR in the present study was comparable to that reported in the prior Japan Cardiovascular Surgery Database and in the real-world data in the US [13]. Especially, the in-hospital death rate in patients who underwent SAVR without concomitant procedure was quite low in this study.

The in-hospital death and stroke rates in the TAVI group in the present study were numerically lower than those reported in the prior Japanese TAVI registry conducted early after introduction of TAVI [14]. Moreover, the in-hospital death rate after TAVI in the present study was lower than those reported in European/US countries [7]. Considering the comparable in-hospital death rate in both the SAVR and TAVI groups, both strategies would be good interventional options for patients with severe AS. Nevertheless, the rates of new-onset atrial fibrillation and major bleeding were much higher in the SAVR group than in the TAVI group consistent with the prior studies [5, 6]. Regarding the echocardiographic data at discharge, the incidence of patient-prosthesis mismatch was lower in the TAVI group than in the SAVR group. The clinical impact of patient-prosthesis mismatch after TAVI has been still controversial [15]. However, several studies demonstrated the impact of moderate or severe patient-prosthesis mismatch after SAVR on worse long-term clinical outcomes [16]. Further follow-up would be warranted to evaluate very long-term clinical outcomes after SAVR and TAVI in terms of valve durability and the impact of patient-prosthesis mismatch. Nevertheless, given its less invasive nature and lower rates of procedural complications, TAVI might be the preferred treatment even in younger patients with severe AS who are currently treated with SAVR.

In contrast with the favorable in-hospital outcomes after SAVR in patients without dialysis, the in-hospital death rate in patients with dialysis was high after SAVR. The high rate of in-hospital death after SAVR in patients with dialysis in the present study was consistent with those in the previous studies [17]. In the present study, we could not evaluate the outcomes after TAVI in patients with dialysis, because TAVI for patients with dialysis was not approved in Japan during the enrollment period. The Society of Thoracic Surgeons (STS)/American College of Cardiology (ACC) TVT (Transcatheter Valve Therapies) registry revealed that patients with dialysis had higher risk of in-hospital death after TAVI than those without dialysis [18]. A large study would be warranted to evaluate clinical outcomes and valve durability in patients with dialysis who underwent TAVI and SAVR.

Regarding patients with BAV, in-hospital death rate and stroke were numerically lower in the SAVR group than in the TAVI group. Nevertheless, considering the large differences in age and patient characteristics between the TAVI and SAVR groups, both SAVR and TAVI would be reasonable options for selected patients with BAV [19]. Further study would be warranted to evaluate clinical outcomes for patients with BAV who underwent TAVI and SAVR.

### Study limitations

There were several limitations in this study. First, among 21 participating centers, TAVI was not available in 11 centers, and SAVR was not available in 1 center. Therefore, the decision on proceeding to AVR and choice between TAVI and SAVR must have been affected by the availability of TAVI and SAVR in the individual centers. Second, we did not perform statistical testing for the comparison of clinical outcomes, because the selection of treatment must have been influcenced by factors other than the measured characteristics, and the low incidences of the clinical events did not allow the appropriate adjustment for the large differences in the baseline characteristics between the SAVR and TAVI groups. Third, TAVI for patients with dialysis was not approved in Japan during the study.

## Conclusions

In this real-world data in Japan, TAVI compared with SAVR was chosen in much older patients with severe AS. In-hospital death rate was numerically lower in the TAVI group than in the SAVR group. Among patients without dialysis, In-hospital death rate in patients was very low and comparable in the two groups.

### Supplementary Information

Below is the link to the electronic supplementary material.

Supplementary file1 (DOC 521 KB)

## References

[1] Schwarz F, Baumann P, Manthey J, Hoffmann M, Schuler G, Mehmel HC, Schmitz W, Kübler W. The effect of aortic valve replacement on survival. Circulation. 1982;66(5):1105–10. 10.1161/01.CIR.66.5.1105. 10.1161/01.cir.66.5.1105 7127696

[2] Leon MB, Smith CR, Mack M, Miller DC, Moses JW, Svensson LG, PARTNER Trial Investigators (2010). Transcatheter aortic-valve implantation for aortic stenosis in patients who cannot undergo surgery. N Engl J Med.

[3] Smith CR, Leon MB, Mack MJ, Miller DC, Moses JW, Svensson LG, PARTNER Trial Investigators (2011). Transcatheter versus surgical aortic-valve replacement in high-risk patients. N Engl J Med.

[4] Leon MB, Smith CR, Mack MJ, Makkar RR, Svensson LG, Kodali SK (2016). Transcatheter or surgical aortic-valve replacement in intermediate-risk patients. N Engl J Med.

[5] Popma JJ, Deeb GM, Yakubov SJ, Mumtaz M, Gada H, O’Hair D (2019). Transcatheter aortic-valve replacement with a self-expanding valve in low-risk patients. N Engl J Med.

[6] Mack MJ, Leon MB, Thourani VH, Makkar R, Kodali SK, Russo M (2019). Transcatheter aortic-valve replacement with a balloon-expandable valve in low-risk patients. N Engl J Med.

[7] Carroll JD, Mack MJ, Vemulapalli S, Herrmann HC, Gleason TG, Hanzel G (2020). STS-ACC TVT registry of Transcatheter aortic valve replacement. J Am Coll Cardiol.

[8] Mori M, Gupta A, Wang Y, Vahl T, Nazif T, Kirtane AJ (2021). Trends in transcatheter and surgical aortic valve replacement among older adults in the United States. J Am Coll Cardiol.

[9] Kamon T, Kaneko H, Kiriyama H, Itoh H, Fujiu K, Kumazawa R (2020). Transcatheter aortic valve implantation and surgical aortic valve replacement for aortic stenosis in Japan - analysis of a nationwide inpatient database. Circ Rep.

[10] Taniguchi T, Morimoto T, Shiomi H, Ando K, Kanamori N, Murata K, CURRENT AS Registry Investigators (2015). Initial surgical versus conservative strategies in patients with asymptomatic severe aortic stenosis. J Am Coll Cardiol.

[11] Takeji Y, Taniguchi T, Morimoto T, Shirai S, Kitai T, Tabata H, et al, (2020) CURRENT AS Registry-2 Investigators. Rationale, Design, and Baseline Characteristics of the CURRENT AS Registry-2. Circ J 86 (11) 1769–1776. 10.1253/circj.CJ-21-1062 35444112

[12] De Sciscio P, Brubert J, De Sciscio M, Serrani M, Stasiak J, Moggridge GD (2017). Quantifying the shift toward transcatheter aortic valve replacement in low-risk patients. Circ Cardiovasc Qual Outcomes.

[13] Thourani VH, Suri RM, Gunter RL, Sheng S, O’Brien SM, Ailawadi G (2015). Contemporary real-world outcomes of surgical aortic valve replacement in 141,905 low-risk, intermediate-risk, and high-risk patients. Ann Thorac Surg.

[14] Takimoto S, Saito N, Minakata K, Shirai S, Isotani A, Arai Y (2016). Favorable clinical outcomes of transcatheter aortic valve implantation in Japanese patients - first report from the post-approval K-TAVI registry. Circ J.

[15] Herrmann HC, Daneshvar SA, Fonarow GC, Stebbins A, Vemulapalli S, Desai ND (2018). Prosthesis-patient mismatch in patients undergoing transcatheter aortic valve replacement: From the STS/ACC TVT registry. J Am Coll Cardiol.

[16] Head SJ, Mokhles MM, Osnabrugge RLJ, Pibarot P, MacK MJ, Takkenberg JJM (2012). The impact of prosthesispatient mismatch on long-term survival after aortic valve replacement: A systematic review and meta-analysis of 34 observational studies comprising 27 186 patients with 133 141 patient-years. Eur Heart J.

[17] Yamauchi T, Yamamoto H, Miyata H, Kobayashi J, Masai T, Motomura N (2020). Surgical aortic valve replacement for aortic stenosis in dialysis patients - analysis of japan cardiovascular surgery database. Circ J.

[18] Szerlip M, Zajarias A, Vemalapalli S, Brennan M, Dai D, Maniar H (2019). Transcatheter aortic valve replacement in patients with end-stage renal disease. J Am Coll Cardiol.

[19] Vincent F, Ternacle J, Denimal T (2021). Transcatheter aortic valve replacement in bicuspid aortic valve stenosis. Circulation.

