# Supplementary material for: In-hospital outcomes after SAVR or TAVI in patients with severe aortic stenosis
Source: Cardiovasc Interv Ther. 2023 Jun 22;39(1):65–73. doi: 10.1007/s12928-023-00942-x (PMC10764526; doi:10.1007/s12928-023-00942-x)
Supplement: Supplementary file 1 — Supplementary file1 (DOC 521 KB) [file 12928_2023_942_MOESM1_ESM.doc]

**SUPPLEMENTARY MATERIAL**

**Table of contents**

**Supplementary Appendix (A-D) 2**

**Supplemental Table 1-13 11**

**Supplemental Figure 1 34**

**Supplemental Appendix A: List of participating centers and investigators in the CURRENT AS Registry-2**

**Cardiology**

Kyoto University Hospital: Takeshi Kimura, Naritatsu Saito, Takao Kato, Hirotoshi Watanabe, Hiroki Shiomi, Toshiaki Toyota, Eri Minamino-Muta, Yasauki Takeji, Tomoya Yoneda, Nao Kaneko

Kokura Memorial Hospital: Kenji Ando, Shinichi Shirai, Tomohiko Taniguchi, Hiroyuki Tabata, Kazuki Kitano

Kobe City Medical Center General Hospital: Yutaka Furukawa, Takeshi Kitai

National Cerebral and Cardiovascular Center: Chisato Izumi, Masashi Amano

Tenri Hospital: Makoto Miyake

Kansai Denryoku Hospital: Katsuhisa Ishii, Shunsuke Usami

Osaka Red Cross Hospital: Tsukasa Inada, Kazuya Nagao

Kitano Hospital: Moriaki Inoko

Japanese Red Cross Wakayama Medical Center: Takashi Tamura, Mamoru Toyofuku

Mitsubishi Kyoto Hospital: Shinji Miki, Takashi Yokomatsu, Masashi Kato, Kenji Nakatsuma

Kurashiki Central Hospital: Kazushige Kadota, Ryosuke Murai, Kohei Osakada

Shiga General Hospital: Shigeru Ikeguchi, Yasutaka Inuzuka

Shimada General Medical Center: Takeshi Aoyama, Norio Kanamori

Shizuoka City Shizuoka　Hospital: Tomoya Onodera, Koichiro Murata

Shizuoka General Hospital: Hiroki Sakamoto, Yasuyo Takeuchi, Tomohisa Tada

Kindai University Nara Hospital: Manabu Shirotani, Hirokazu Mitsuoka

Kishiwada City Hospital: Mitsuo Matsuda, Masayasu Izuhara

Hyogo Prefectural Amagasaki General Medical Center: Yukihito Sato, Hiroyuki Nakayama

National Hospital Organization Kyoto Medical Center: Masaharu Akao, Mitsuru Ishii

Koto Memorial Hospital: Hiroshi Mabuchi, Masahiro Kimura

Japanese Red Cross Otsu Hospital: Kazuaki Kaitani, Noriko Nakazeki, Marie Okabayashi

**Cardiovascular surgery**

Kyoto University Hospital: Kenji Minatoya, Kazuhiro Yamazaki

Kokura Memorial Hospital: Nobuhisa Ohno, Yoshio Arai

Kobe City Medical Center General Hospital: Tadaaki Koyama

National Cerebral and Cardiovascular Center: Tomoyuki Fujita, Satsuki Fukushima

Tenri Hospital: Atsushi Iwakura, Kyokun Uehara

Kansai Denryoku Hospital: Etsuro Suenaga

Osaka Red Cross Hospital: Shogo Nakayama, Michihito Nonaka

Kitano Hospital: Michiya Hanyu

Japanese Red Cross Wakayama Medical Center: Naoki Kanemitsu, Hisashi Sakaguchi

Mitsubishi Kyoto Hospital: Hitoshi Okabayashi, Jiro Ezaki

Kurashiki Central Hospital: Tatsuhiko Komiya, Jota Nakano, Shingo Hirao

Shiga General Hospital: Yamada Tomoyuki, Kazuhiko Katsuyama

Shizuoka City Shizuoka　Hospital: Fumio Yamazaki, Hiroshi Mitsuoka, Masanao Nakai

Shizuoka General Hospital: Hiroshi Tsuneyoshi

Kindai University Nara Hospital: Noboru Nishiwaki

Kishiwada City Hospital: Onoe Nasahiko, Kosuke Fujii

Hyogo Prefectural Amagasaki General Medical Center: Nobushige Tamura, OkadaTatsuji

National Hospital Organization Kyoto Medical Center: Kotaro Shiraga, Tsuyoshi Kataoka

Koto Memorial Hospital: Toshihiro Kawahira, Fumihiro Miyashita

Japanese Red Cross Otsu Hospital: Kouji Ueyama

**Supplemental Appendix B: Definitions of the clinical events**

Death was regarded as cardiovascular in origin unless obvious non-cardiovascular causes could be identified. Sudden death was defined as unexplained death in previously stable patients. Any death during the hospitalization for aortic valve replacement or transcatheter aortic valve implantation was regarded as aortic valve procedure-related death. Aortic valve-related death included aortic valve procedure-related death, sudden death, and death due to heart failure related to aortic stenosis. Heart failure hospitalization was defined as hospitalization due to worsening heart failure requiring intravenous drug therapy

Stroke was defined as duration of a focal or global neurological deficit ≥24 h; OR <24 h if available neuroimaging documents a new haemorrhage or infarct; OR the neurological deficit results in death. Ischaemic stroke was defined as an acute episode of focal cerebral, spinal, or retinal dysfunction caused by infarction of the central nervous system tissue. Haemorrhagic stroke was defined as an acute episode of focal or global cerebral or spinal dysfunction caused by intraparenchymal, intraventricular, or subarachnoid haemorrhage. Disabling stroke was defined as an modified Rankin Scale (mRS) score of 2 or more at 90 days and an increase in at least one mRS category from an individual’s pre-stroke baseline. Non-disabling stroke was defined an mRS score of <2 at 90 days or one that does not result in an increase in at least one mRS category from an individual’s pre-stroke baseline.^1^

Peri-procedural MI (≤72 h after the index procedure) was defined as^1^

・New ischaemic symptoms (e.g. chest pain or shortness of breath), or new ischaemic signs (e.g. ventricular arrhythmias, new or worsening heart failure, new ST-segment changes, haemodynamic instability, new pathological Q-waves in at least two contiguous leads, imaging evidence of new loss of viable myocardium or new wall motion abnormality) AND

・Elevated cardiac biomarkers (preferable CK-MB) within 72 h after the index procedure, consisting of at least one sample post-procedure with a peak value exceeding 15× as the upper reference limit for troponin or 5× for CK-MB.a If cardiac biomarkers are increased at baseline (>99th percentile), a further increase in at least 50% post-procedure is required AND the peak value must exceed the previously stated limit.

Spontaneous MI (>72 h after the index procedure) as any one of the following criteria^1^:

・Detection of rise and/or fall of cardiac biomarkers (preferably

troponin) with at least one value above the 99th percentile URL,

together with the evidence of myocardial ischaemia with at least

one of the following:

・Symptoms of ischaemia

・ECG changes indicative of new ischaemia [new ST-T changes or new left bundle branch block (LBBB)]

・New pathological Q-waves in at least two contiguous leads

・Imaging evidence of a new loss of viable myocardium or new wall motion abnormality

・Sudden, unexpected cardiac death, involving cardiac arrest, often with symptoms suggestive of myocardial ischaemia, and accompanied by presumably new ST elevation, or new LBBB, and/or evidence of fresh thrombus by coronary angiography

and/or at autopsy, but death occurring before blood samples could be obtained, or at a time before the appearance of cardiac biomarkers in the blood.

・Pathological findings of an acute myocardial infarction.

Myocardial infarction (MI) during the follow-up period was defined in accordance with the universal MI guidelines.^2^

The Valve Academic Research Consortium (VARC)- 3 bleeding was defined according to Bleeding Academic Research Consortium (BARC) definition. ^3,4^ Major bleeding was defined as VARC-3 Type 2, 3, and 4 in this study.

Overt bleeding was defined that fulfils one of the following criteria:

**Type 1**

• Overt bleeding that does not require surgical or percutaneous intervention, but does require medical intervention by a health care professional, leading to hospitalization, an increased level of care, or medical evaluation (BARC 2)

• Overt bleeding that requires a transfusion of 1 unit* of whole blood/red blood cells (BARC 3a)

**Type 2**

• Overt bleeding that requires a transfusion of 2–4 units of whole blood/red blood cells (BARC 3a)

• Overt bleeding associated with a haemoglobin drop of >3 g/dL (>1.86 mmol/L) but <5 g/d (<3.1 mmol/L) (BARC 3a)

**Type 3**

• Overt bleeding in a critical organ, such as intracranial, intraspinal, intraocular, pericardial (associated with haemodynamic compromise/tamponade and necessitating intervention), or intramuscular with compartment syndrome (BARC 3b, BARC 3c)

• Overt bleeding causing hypovolemic shock or severe hypotension (systolic blood pressure <90 mmHg lasting >30 min and not responding to volume resuscitation) or requiring vasopressors or surgery (BARC 3b)

• Overt bleeding requiring reoperation, surgical exploration, or re-intervention for the purpose of controlling bleeding (BARC 3b, BARC 4)

• Post-thoracotomy chest tube output ≥2 L within a 24-h period (BARC 4)

• Overt bleeding requiring a transfusion of ≥5 units of whole blood/red blood cells (BARC 3a)

• Overt bleeding associated with a haemoglobin drop ≥5 g/dL (≥3.1 mmol/L) (BARC 3b).

**Type 4**

• Overt bleeding leading to death (BARC 5).

*Units of blood transfusion are described as Japanese unit.

Newly diagnosed AF was defined as presumably newly developed AF documented during the index procedural hospitalization. Pacemaker implantation was defined as newly implanted pacemaker from any cause after the procedure.

Major vascular complication was defined as meeting one of the following^3^:

・Aortic dissection or aortic rupture,

・Vascular (arterial or venous) injury (perforation, rupture, dissection, stenosis, ischaemia, arterial or venous thrombosis including pulmonary embolism, arteriovenous fistula, pseudoaneurysm, haematoma, retroperitoneal haematoma, infection) or compartment syndrome resulting in death, VARC type 2　bleeding, limb or visceral ischaemia, or irreversible neurologic impairment

・Distal embolization (non-cerebral) from a vascular source resulting in death, amputation, limb or visceral ischaemia, or irreversible end-organ damage

・Unplanned endovascular or surgical intervention resulting in death, VARC type $2 bleeding, limb or visceral ischaemia, or irreversible neurologic　impairment

・Closure device failure resulting in death, VARC type 2 bleeding, limb or visceral ischaemia, or irreversible neurologic impairment

**Supplemental Appendix C: List of the clinical event committee members in the CURRENT AS Registry-2**

Hiroki Shiomi (Kyoto University Hospital), Hirotoshi Watanabe (Kyoto University Hospital),

Kenji Nakatsuma (Mitsubishi Kyoto Hospital)

**Supplemental Table1. Number of cases in each participating center.**

| Participating center | Number of enrolled patients | | |
| --- | --- | --- | --- |
|  | Entire study population | SAVR group | TAVI group |
| A | 9 | | |
|  | 7 | 6 | 1 |
| B | 32 | | |
|  | 21 | 15 | 6 |
| C |  | 209 |  |
|  | 113 | 34 | 79 |
| D | 6 | | |
|  | 3 | 2 | 1 |
| E | 34 | | |
|  | 22 | 22 | 0 |
| F | 172 | | |
|  | 146 | 70 | 76 |
| G | 78 | | |
|  | 47 | 14 | 33 |
| H | 100 | | |
|  | 32 | 21 | 11 |
| I | 156 | | |
|  | 29 | 19 | 10 |
| J | 3 | | |
|  | 3 | 3 | 0 |
| K | 1149 | | |
|  | 684 | 171 | 513 |
| L | 44 | | |
|  | 22 | 10 | 12 |
| M | 206 | | |
|  | 179 | 44 | 135 |
| N | 251 | | |
|  | 114 | 47 | 67 |
| O | 349 | | |
|  | 115 | 40 | 75 |
| P | 91 | | |
|  | 20 | 16 | 4 |
| Q | 7 | | |
|  | 1 | 0 | 1 |
| R | 107 | | |
|  | 66 | 6 | 60 |
| S | 172 | | |
|  | 36 | 27 | 9 |
| T | 102 | | |
|  | 44 | 7 | 37 |
| U | 38 | | |
|  | 10 | 6 | 4 |

**Supplemental Table 2. Full items of p**atient characteristics

|  | **SAVR group** | **TAVI group** | **P value** | **N of patients evaluated** |
| --- | --- | --- | --- | --- |
|  | **(N=580)** | **(N=1134)** |  | **(N=1714)** |
| **(A) Clinical characteristics** |  |  |  |  |
| Age (years) | 73.6±7.6 | 84.4±5.6 | <0.001 | 1714 |
| Age ≥80 years | 115 (20%) | 962 (85%) | <0.001 | 1714 |
| Men | 304 (52%) | 396 (35%) | <0.001 | 1714 |
| Body mass index (kg/m^2^) | 23.2±3.8 | 22.7±3.7 | 0.01 | 1714 |
| Body mass index <22.0 kg/m^2^ | 231 (40%) | 516 (46%) | 0.02 | 1714 |
| BSA, m^2^ | 1.6±0.2 | 1.5±0.2 | <0.001 | 1714 |
| Systolic blood pressure, mmHg | 132±22 | 133±22 | 0.84 | 1321 |
| Diastolic blood pressure, mmHg | 71±13 | 69±13 | <0.001 | 1320 |
| Heart rate, beat per minutes | 73±14 | 73±14 | 0.53 | 1149 |
| Hypertension | 436 (75%) | 956 (84%) | <0.001 | 1714 |
| Current smoking | 42 (7.2%) | 31 (2.7%) | <0.001 | 1714 |
| Dyslipidemia | 310 (53%) | 588 (52%) | 0.53 | 1714 |
| Diabetes mellitus | 177 (31%) | 303 (27%) | 0.10 | 1714 |
| on insulin therapy | 28 (4.8%) | 40 (3.5%) | 0.19 | 1714 |
| Prior myocardial infarction | 24 (4.1%) | 70 (6.2%) | 0.08 | 1714 |
| Prior PCI | 63 (11%) | 156 (14%) | 0.09 | 1714 |
| Prior CABG | 4 (0.7%) | 36 (3.2%) | 0.001 | 1714 |
| Prior open heart surgery | 14 (2.4%) | 49 (4.3%) | 0.047 | 1714 |
| Prior symptomatic stroke | 53 (9.1%) | 158 (14%) | 0.004 | 1714 |
| Atrial fibrillation or flutter | 104 (18%) | 246 (22%) | 0.07 | 1714 |
| Aortic and/or peripheral vascular disease | 44 (7.6%) | 68 (6.0%) | 0.21 | 1714 |
| eGFR (mL/min/1.73m^2^) | 50.2±26.6 | 51.4±19.0 | 0.28 | 1689 |
| eGFR <30 mL/min/1.73m^2^ not on dialysis | 28 (4.8%) | 139 (12%) | <0.001 | 1714 |
| Serum creatinine | 0.9 (0.7-1.4) | 0.9 (0.7-1.2) | 0.005 | 1689 |
| Creatinine level >2 mg/dl or dialysis | 117 (20%) | 45 (4.0%) | <0.001 | 1714 |
| Dialysis | 105 (18%) | 2 (0.2%) | <0.001 | 1714 |
| Anemia | 233 (40%) | 719 (63%) | <0.001 | 1714 |
| Liver cirrhosis (Child B or C) | 3 (0.5%) | 12 (1.1%) | 0.26 | 1714 |
| Malignancy | 98 (17%) | 230 (20 %) | 0.09 | 1714 |
| Malignancy currently under treatment | 22 (3.8%) | 69 (6.1%) | 0.045 | 1714 |
| Chest wall irradiation | 5 (0.9%) | 8 (0.7%) | 0.72 | 1714 |
| Immunosuppressive therapy | 22 (3.8%) | 70 (6.2%) | 0.04 | 1714 |
| Chronic lung disease | 180 (31%) | 381 (34%) | 0.28 | 1714 |
| Chronic lung disease (moderate or severe) | 22 (3.8%) | 106 (9.3%) | <0.001 | 1714 |
| Coronary artery disease | 226 (39%) | 448 (40%) | 0.83 | 1714 |
| Clinical Frailty Scale |  |  | <0.001 | 1714 |
| 1-3 | 423 (73%) | 550 (49%) |  |  |
| 4-6 | 151 (26%) | 531 (47%) |  |  |
| 7-9 | 6 (1.0%) | 53 (4.7%) |  |  |
| STS PROM, % | 2.9 (1.8-4.5) | 4.6 (3.3-6.3) | <0.001 | 1714 |
| EuroSCORE II, % | 2.7 (1.5-5.1) | 3.6 (2.6-4.8) | <0.001 | 1714 |
| Logistic EuroSCORE, % | 7.1 (4.8-11.8) | 12.1 (9.0-18.7) | <0.001 | 1714 |
| BNP (pg/ml) | 138 (56-432) | 187 (75 -428) | 0.005 | 1539 |
| NT-proBNP (pg/ml) | 1107 (310-6528) | 1058 (472-3151) | 0.93 | 182 |
| **(B) Presentation** |  |  |  |  |
| **Etiology** |  |  | <0.001 |  |
| Degenerative | 440 (76%) | 1093 (96%) |  | 1714 |
| Congenital (unicuspid, bicuspid, or quadricuspid) | 125 (22%) | 34 (3.0%) |  | 1714 |
| Rheumatic | 11 (1.9%) | 7 (0.6%) |  | 1714 |
| Infective endocarditis | 3 (0.5%) | 0 (0.0%) |  | 1714 |
| Other | 1 (0.2%) | 0 (0.0%) |  | 1714 |
| **Symptoms** |  |  |  |  |
| AS related symptoms | 441 (76%) | 946 (83%) | <0.001 | 1714 |
| Chest pain | 95 (22%) | 129 (14%) | <0.001 | 1714 |
| Syncope | 40 (9.1%) | 114 (12%) | 0.10 | 1714 |
| Heart failure | 362 (82%) | 837 (89%) | 0.001 | 1714 |
| NYHA class |  |  | 0.052 |  |
| II | 256 (71%) | 532 (64%) |  | 1714 |
| III | 81 (22%) | 239 (29%) |  | 1714 |
| IV | 25 (6.9%) | 66 (7.9%) |  | 1714 |
| **(C) Medications at index echocardiography** |  |  |  |  |
| Antiplatelet therapy |  |  |  |  |
| Aspirin | 139 (24%) | 281 (25%) | 0.71 | 1714 |
| Thienopyridine |  |  |  |  |
| Clopidogrel | 47 (8.1%) | 125 (11%) | 0.06 | 1714 |
| Prasugrel | 6 (1.0%) | 12 (1.1%) | 0.96 | 1714 |
| Ticlopidine | 0 (0.0%) | 4 (0.4%) | 0.15 | 1714 |
| Statins | 274 (47%) | 500 (44%) | 0.21 | 1714 |
| Beta-blockers | 142 (25%) | 314 (28%) | 0.15 | 1714 |
| ACE inhibitors/ARB | 272 (47%) | 591 (52%) | 0.04 | 1714 |
| Calcium channel blockers | 270 (47%) | 634 (56%) | <0.001 | 1714 |
| Warfarin | 42 (7.2%) | 66 (5.8%) | 0.25 | 1714 |
| DOAC | 48 (8.3%) | 188 (17%) | <0.001 | 1714 |
| Proton pump inhibitors | 213 (37%) | 509 (45%) | 0.001 | 1714 |
| H2 blockers | 34 (5.9%) | 68 (6.0%) | 0.91 | 1714 |
| Oral hypoglycemic agents | 131 (23%) | 227 (20%) | 0.22 | 1714 |

Anemia was defined as serum hemoglobin <12 g/dl for women or <13 g/dl for men.

The patients

Coronary artery disease was defined to be present when meeting at least one of the following criteria: a history of PCI or CABG, a history of myocardial infarction, presentation as myocardial infarction at baseline, or angiographically confirmed coronary artery disease at baseline.

AVR=aortic valve replacement, TAVI=transcatheter aortic valve implantation; SAVR=surgical aortic valve replacement; BSA=body surface area; PCI=percutaneous coronary intervention; CABG=coronary artery bypass grafting; STS=society of thoracic surgeons; PROM=predicted risk of mortality; NYHA=New York Heart Association; ACE=angiotensin-converting enzyme; ARB=angiotensin receptor blocker; DOAC=direct oral anticoagulant; H2= histamine type 2 receptor.

**Supplemental Table 3.** Echocardiographic characteristics

|  | **SAVR group** | **TAVI group** | **P value** | **N of patients evaluated** |
| --- | --- | --- | --- | --- |
|  | **(N=580)** | **(N=1134)** |  |  |
| Vmax (m/s) | 4.5±0.7 | 4.5±0.8 | 0.58 | 1714 |
| Vmax >4 m/s | 439 (76%) | 864 (76%) | 0.82 | 1714 |
| Vmax >4.5 m/s | 251 (43%) | 507 (45%) | 0.57 | 1714 |
| Vmax >5 m/s | 126 (22%) | 269 (24%) | 0.35 | 1714 |
| Mean aortic PG (mm Hg) | 47.7±17.9 | 48.9±17.5 | 0.16 | 1710 |
| Mean aortic PG >40mmHg | 360 (62%) | 763 (68%) | 0.03 | 1710 |
| AVA (cm^2^) | 0.7±0.2 | 0.7±0.2 | <0.001 | 1714 |
| AVA <1.0 cm^2^ | 547 (95%) | 1107 (98%) | 0.001 | 1714 |
| AVA index, cm^2^/m^2^ | 0.5±0.1 | 0.5±0.1 | 0.97 | 1714 |
| LVEF, % | 60.3±11.5 | 60.5±10.6 | 0.72 | 1714 |
| <40% | 45 (7.8%) | 70 (6.2%) | 0.21 | 1714 |
| <50% | 90 (16%) | 173 (15%) | 0.89 | 1714 |
| <60% | 189 (33%) | 372 (33%) | 0.93 | 1714 |
| Stroke volume index (ml/m^2^) | 46±11 | 46±12 | 0.24 | 1708 |
| Stroke volume index ≤35 ml/m^2^ | 101 (18%) | 204 (18%) | 0.80 | 1708 |
| Eligibility for severe AS |  |  |  | 1714 |
| High-gradient AS | 452 (78%) | 878 (77%) | 0.81 |  |
| Low-gradient AS | 128 (22%) | 256 (23%) | - |  |
| Low-flow low-gradient AS with reduced LVEF | 22 (3.8%) | 41 (3.6%) | 0.85 |  |
| Low-flow low-gradient AS with preserved LVEF | 23 (4.0%) | 44 (3.9%) | 0.93 |  |
| Normal-flow low-gradient AS with preserved LVEF | 69 (12%) | 137 (12%) | 0.91 |  |
| Normal-flow low-gradient AS with reduced LVEF | 14 (2.4%) | 34 (3.0%) | 0.49 |  |
| LV end-diastolic diameter (mm) | 47±7 | 44±6 | <0.001 | 1713 |
| LV end-systolic diameter (mm) | 31±7 | 30±7 | <0.001 | 1700 |
| IVST in diastole (mm) | 11±2 | 11±2 | 0.33 | 1697 |
| PWT in diastole (mm) | 11±2 | 11±2 | 0.67 | 1697 |
| LVMI (g/m^2^) | 114±33 | 112±29 | 0.19 | 1697 |
| High LVMI (Men: > 115, and Women: > 95) | 325 (56%) | 641 (57%) | 0.59 | 1697 |
| Any combined valvular disease (Moderate or severe) | 161 (28%) | 258 (23%) | 0.02 | 1714 |
| Moderate or severe AR | 82 (14%) | 88 (7.8%) | <0.001 | 1714 |
| Moderate or severe MS | 23 (4.0%) | 29 (2.6%) | 0.11 | 1714 |
| Moderate or severe MR | 66 (11%) | 124 (11%) | 0.78 | 1714 |
| Moderate or severe TR | 47 (8.1%) | 84 (7.4%) | 0.61 | 1714 |
| TR pressure gradient ≥40 mm Hg | 46 (7.9%) | 131 (12%) | 0.02 | 1714 |

AVA was measured by using equation of continuity.

High gradient AS was defined as V_max_>4.0 m/s or mean aortic PG>40 mmHg, while low gradient AS was defined as V_max_ ≤ 4.0 m/s and mean aortic PG ≤ 40 mmHg.

Low-flow low-gradient AS with reduced LVEF was defined as patients who met all the following criteria: AVA <1 cm^2^, mPG ≤ 40mmHg, Vmax≤ 4.0m/s, stroke volume index ≤ 35mL/m^2^, and LVEF <50%.

Low-flow low-gradient AS with preserved LVEF was defined as patients who met all the following criteria: AVA <1 cm^2^, mPG ≤ 40mmHg, Vmax≤ 4.0m/s, stroke volume index ≤ 35mL/m^2^, and LVEF ≥50%.

Normal-flow low-gradient AS was defined as patients who met all the following criteria: AVA <1 cm^2^, mPG ≤ 40mmHg, Vmax≤ 4.0m/s, and stroke volume index >35mL/m^2^.

Five patients were enrolled with Vmax=4.0, mPG ≤ 40mmHg, and AVA>1.0 (protocol violation). These patients were regarded as having high gradient AS.

V_max_=peak aortic jet velocity; PG=pressure gradient; AVA=aortic valve area; AS=aortic stenosis; LV=left ventricular; LVEF=left ventricular ejection fraction; LVMI=left ventricular mass index; IVST=interventricular septum thickness; PWT=posterior wall thickness; AR=aortic regurgitation; MS=mitral stenosis; MR=mitral regurgitation; TR=tricuspid regurgitation.

**Supplemental Table 4**. Patient characteristics according to the age categories

|  | **AGE<75** | |  | **75**≤**AGE<80** | |  | **AGE**≥**80** | |  |
| --- | --- | --- | --- | --- | --- | --- | --- | --- | --- |
|  | **SAVR group** | **TAVI group** | **P value** | **SAVR group** | **TAVI group** | **P value** | **SAVR group** | **TAVI group** | **P value** |
|  | **(N=285)** | **(N=53)** |  | **(N=180)** | **(N=119)** |  | **(N=115)** | **(N=962)** |  |
| **(A) Clinical characteristics** | |  |  |  |  |  |  |  |  |
| Age (years) | 67.8±6.4 | 70.5±5.2 | 0.005 | 77.0±1.4 | 77.5±1.3 | 0.002 | 82.7±2.1 | 86.0±4.0 | <0.001 |
| Men | 165 (58%) | 31 (59%) | 0.94 | 79 (44%) | 56 (47%) | 0.59 | 60 (52%) | 309 (32%) | <0.001 |
| Body mass index (kg/m^2^) | 23.4±4.1 | 23.7±3.7 | 0.70 | 23.4±3.7 | 24.2±4.2 | 0.07 | 22.3±3.1 | 22.5±3.5 | 0.68 |
| Body mass index <22.0 kg/m^2^ | 111 (39%) | 20 (38%) | 0.87 | 67 (37%) | 36 (30%) | 0.21 | 53 (46%) | 460 (48%) | 0.73 |
| BSA, m^2^ | 1.7±0.2 | 1.6±0.2 | 0.65 | 1.6±0.2 | 1.6±0.2 | 0.53 | 1.5±0.2 | 1.5±0.2 | <0.001 |
| Systolic blood pressure, mmHg | 131±23 | 129±23 | 0.58 | 133±21 | 132±21 | 0.79 | 134±19 | 133±22 | 0.67 |
| Diastolic blood pressure, mmHg | 73±13 | 71±14 | 0.36 | 70±13 | 70±12 | 0.72 | 69±14 | 68±13 | 0.39 |
| Heart rate, beat per minutes | 74±14 | 75±13 | 0.65 | 72±14 | 72±14 | 0.66 | 73±12 | 72±14 | 0.62 |
| Hypertension | 195 (68%) | 39 (74%) | 0.45 | 144 (80%) | 103 (86%) | 0.14 | 97 (84%) | 814 (85%) | 0.94 |
| Current smoking | 31 (11%) | 8 (15%) | 0.38 | 8 (4.4%) | 9 (7.6%) | 0.25 | 3 (2.6%) | 14 (1.5%) | 0.35 |
| Dyslipidemia | 145 (51%) | 27 (51%) | 0.99 | 107 (59%) | 66 (56%) | 0.50 | 58 (50%) | 495 (52%) | 0.84 |
| Diabetes mellitus | 91 (32%) | 21 (40%) | 0.28 | 59 (33%) | 42 (35%) | 0.65 | 27 (24%) | 240 (25%) | 0.73 |
| on insulin therapy | 16 (5.6%) | 4 (7.5%) | 0.58 | 8 (4.4%) | 3 (2.5%) | 0.39 | 4 (3.5%) | 33 (3.4%) | 0.98 |
| Prior myocardial infarction | 11 (3.9%) | 4 (7.5%) | 0.23 | 10 (5.6%) | 8 (6.7%) | 0.68 | 3 (2.6%) | 58 (6.0%) | 0.13 |
| Prior PCI | 27 (9.5%) | 8 (15%) | 0.22 | 21 (12%) | 17 (14%) | 0.51 | 15 (13%) | 131 (14%) | 0.87 |
| Prior CABG | 3 (1.1%) | 8 (15%) | <0.001 | 1 (0.6%) | 6 (5.0%) | 0.01 | 0 (0.0%) | 22 (2.3%) | 0.10 |
| Prior open heart surgery | 10 (3.5%) | 11 (21%) | <0.001 | 2 (1.1%) | 8 (6.7%) | 0.008 | 2 (1.7%) | 30 (3.1%) | 0.41 |
| Prior symptomatic stroke | 27 (9.5%) | 11 (21%) | 0.02 | 18 (10%) | 22 (19%) | 0.04 | 8 (7.0%) | 125 (13.0%) | 0.06 |
| Atrial fibrillation or flutter | 39 (14%) | 6 (11%) | 0.64 | 43 (24%) | 25 (21%) | 0.56 | 22 (19%) | 214 (22%) | 0.46 |
| Aortic and/or peripheral vascular disease | 17 (6.0%) | 5 (9.4%) | 0.35 | 17 (9.4%) | 8 (6.7%) | 0.41 | 10 (8.7%) | 55 (5.7%) | 0.21 |
| eGFR (mL/min/1.73m^2^) | 51.9±28.5 | 62.6±17.9 | 0.009 | 51.3±22.8 | 56.6±22.8 | 0.06 | 44.1±26.6 | 50.2±18.2 | 0.002 |
| eGFR <30 mL/min/1.73m^2^ not on dialysis | 9 (3.2%) | 2 (3.8%) | 0.82 | 11 (6.1%) | 11 (9.2%) | 0.31 | 8 (7.0%) | 126 (13%) | 0.06 |
| Serum creatinine | 0.9 (0.7-1.5) | 0.8 (0.7-1.0) | 0.04 | 0.9 (0.7-1.2) | 0.9 (0.7-1.2) | 0.59 | 1.0 (0.8-1.6) | 0.9 (0.7-1.2) | <0.001 |
| Creatinine level >2 mg/dl or dialysis | 64 (23%) | 0 (0.0%) | <0.001 | 27 (15%) | 5 (4.2%) | 0.003 | 26 (23%) | 40 (4.2%) | <0.001 |
| Dialysis | 58 (20%) | 0 (0.0%) | <0.001 | 22 (12%) | 0 (0.0%) | <0.001 | 25 (22%) | 2 (0.2%) | <0.001 |
| Anemia | 91 (32%) | 26 (49%) | 0.02 | 77 (43%) | 55 (46%) | 0.56 | 65 (57%) | 638 (66%) | 0.04 |
| Liver cirrhosis (Child B or C) | 2 (0.7%) | 2 (3.8%) | 0.06 | 1 (0.6%) | 2 (1.7%) | 0.34 | 0 (0.0%) | 8 (0.8%) | 0.33 |
| Malignancy | 44 (15%) | 22 (42%) | <0.001 | 34 (19%) | 24 (20%) | 0.78 | 20 (17%) | 184 (19%) | 0.65 |
| Malignancy currently under treatment | 8 (2.8%) | 13 (25%) | <0.001 | 10 (5.6%) | 9 (7.6%) | 0.49 | 4 (3.5%) | 47 (4.9%) | 0.50 |
| Chest wall irradiation | 1 (0.4%) | 1 (1.9%) | 0.18 | 3 (1.7%) | 1 (0.8%) | 0.54 | 1 (0.9%) | 6 (0.6%) | 0.76 |
| Immunosuppressive therapy | 12 (4.2%) | 9 (17%) | <0.001 | 8 (4.4%) | 11 (9.2%) | 0.10 | 2 (1.7%) | 50 (5.2%) | 0.10 |
| Chronic lung disease | 77 (27%) | 20 (38%) | 0.11 | 63 (35%) | 46 (39%) | 0.52 | 40 (35%) | 315 (33%) | 0.66 |
| Chronic lung disease (moderate or severe) | 7 (2.5%) | 7 (13%) | <0.001 | 9 (5.0%) | 15 (13%) | 0.02 | 6 (5.2%) | 84 (8.7%) | 0.20 |
| Coronary artery disease | 93 (33%) | 23 (43%) | 0.13 | 72 (40%) | 45 (38%) | 0.71 | 61 (53%) | 380 (40%) | 0.005 |
| Clinical Frailty Scale |  |  | <0.001 |  |  | 0.048 |  |  | 0.60 |
| 1-3 | 235 (83%) | 33 (62%) |  | 128 (71%) | 70 (59%) |  | 60 (52%) | 447 (47%) |  |
| 4-6 | 49 (17%) | 17 (32%) |  | 51 (28%) | 46 (39%) |  | 51 (44%) | 468 (49%) |  |
| 7-9 | 1 (0.4%) | 3 (5.7%) |  | 1 (0.6%) | 3 (2.5%) |  | 4 (3.5%) | 47 (4.9%) |  |
| STS PROM, % | 2.1 (1.3-3.5) | 2.6 (1.6-3.5) | 0.21 | 3.1 (2.5-4.6) | 2.9 (2.1-4.3) | 0.054 | 4.5 (3.3-7.0) | 4.9 (3.6-6.6) | 0.40 |
| EuroSCORE II, % | 2.0 (1.2-3.8) | 2.0 (1.2-3.6) | 0.82 | 2.7 (1.8-5.7) | 2.4 (1.5-3.3) | 0.001 | 4.4 (2.9-7.1) | 3.8 (2.8-5.0) | 0.007 |
| Logistic EuroSCORE, % | 5.1 (3.3-9.2) | 6.6 (4.2-12.3) | 0.04 | 7.5 (5.8-12.7) | 7.0 (6.2-13.0) | 0.68 | 11.0 (7.9-17.2) | 12.8 (10.1-19.9) | 0.001 |
| BNP (pg/ml) | 117 (44-407) | 77 (44-299) | 0.44 | 146 (61-371) | 111 (52-204) | 0.07 | 218 (78-590) | 210 (84-459) | 0.60 |
| NT-proBNP (pg/ml) | 1793 (277-11734) | 496 (171-935) | 0.19 | 513 (232-4108) | 879 (417-3951) | 0.39 | 2297 (582-6658) | 1197 (484-3172) | 0.32 |
| **(B) Presentation** | |  |  |  |  |  |  |  |  |
| **Etiology** |  |  | 0.14 |  |  | 0.41 |  |  | 0.001 |
| Degenerative | 179 (63%) | 43 (81%) |  | 157 (87%) | 111 (93%) |  | 105 (91%) | 939 (98%) |  |
| Congenital (unicuspid, bicuspid, or quadricuspid) | 98 (34%) | 9 (17%) |  | 18 (10%) | 6 (5.0%) |  | 9 (7.8%) | 19 (2.0%) |  |
| Rheumatic | 6 (2.1%) | 1 (1.9%) |  | 4 (2.2%) | 2 (1.7%) |  | 1 (0.9%) | 4 (0.4%) |  |
| Infective endocarditis | 1 (0.4%) | 0 (0.0%) |  | 1 (0.6%) | 0 (0.0%) |  | 0 (0.0%) | 0 (0.0%) |  |
| Other | 1 (0.4%) | 0 (0.0%) |  | 0 (0.0%) | 0 (0.0%) |  | 0 (0.0%) | 0 (0.0%) |  |
| **Symptoms** |  |  |  |  |  |  |  |  |  |
| AS related symptoms | 206 (72%) | 42 (79%) | 0.29 | 139 (77%) | 93 (78%) | 0.85 | 96 (84%) | 811 (84%) | 0.82 |
| Chest pain | 39 (19%) | 9 (21%) | 0.71 | 26 (19%) | 20 (22%) | 0.60 | 30 (31%) | 100 (12%) | <0.001 |
| Syncope | 17 (8.3%) | 6 (14%) | 0.22 | 12 (8.6%) | 9 (9.7%) | 0.79 | 11 (12%) | 99 (12%) | 0.83 |
| Heart failure | 173 (84%) | 37 (88%) | 0.50 | 115 (83%) | 76 (82%) | 0.84 | 74 (77%) | 724 (89%) | 0.001 |
| NYHA class |  |  | 0.046 |  |  | 0.30 |  |  | 0.68 |
| II | 121 (70%) | 25 (68%) |  | 85 (74%) | 55 (72%) |  | 50 (68%) | 452 (62%) |  |
| III | 40 (23%) | 5 (14%) |  | 22 (19%) | 19 (25%) |  | 19 (26%) | 215 (30%) |  |
| IV | 12 ( 6.9%) | 7 (19%) |  | 8 (7.0%) | 2 (2.6%) |  | 5 (6.8%) | 57 (7.9%) |  |
| **(C) Medications at index echocardiography** | | | |  |  |  |  |  |  |
| Antiplatelet therapy | |  |  |  |  |  |  |  |  |
| Aspirin | 59 (21%) | 15 (28%) | 0.22 | 44 (24%) | 31 (26%) | 0.75 | 36 (31%) | 235 (24%) | 0.11 |
| Thienopyridine | |  |  |  |  |  |  |  |  |
| Clopidogrel | 20 (7.0%) | 5 (9.4%) | 0.54 | 19 (11%) | 19 (16%) | 0.17 | 8 (7.0%) | 101 (11%) | 0.23 |
| Prasugrel | 4 (1.4%) | 2 (3.8%) | 0.23 | 2 (1.1%) | 2 (1.7%) | 0.68 | 0 (0.0%) | 8 (0.8%) | 0.33 |
| Ticlopidine | 0 (0.0%) | 0 (0.0%) | - | 0 (0.0%) | 0 (0.0%) | - | 0 (0.0%) | 4 (0.4%) | 0.49 |
| Statins | 127 (45%) | 25 (47%) | 0.73 | 95 (53%) | 63 (53%) | 0.98 | 52 (45%) | 412 (43%) | 0.63 |
| Beta-blockers | 74 (26%) | 17 (32%) | 0.36 | 44 (24%) | 36 (30%) | 0.27 | 24 (21%) | 261 (27%) | 0.15 |
| ACE inhibitors/ARB | 124 (44%) | 24 (45%) | 0.81 | 92 (51%) | 70 (59%) | 0.19 | 56 (49%) | 497 (52%) | 0.55 |
| Calcium channel blockers | 116 (41%) | 27 (51%) | 0.17 | 89 (49%) | 67 (56%) | 0.25 | 65 (57%) | 540 (56%) | 0.94 |
| Warfarin | 20 (7.0%) | 1 (1.9%) | 0.16 | 11 (6.1%) | 11 (9.2%) | 0.31 | 11 (9.6%) | 54 (5.6%) | 0.09 |
| DOAC | 16 (5.6%) | 6 (11%) | 0.12 | 23 (13%) | 16 (13%) | 0.87 | 9 (7.8%) | 166 (17%) | 0.01 |
| Proton pump inhibitors | 86 (30%) | 28 (53%) | 0.001 | 63 (35%) | 56 (47%) | 0.04 | 64 (56%) | 425 (44%) | 0.02 |
| H2 blockers | 17 (6.0%) | 0 (0.0%) | 0.07 | 11 (6.1%) | 6 (5.0%) | 0.70 | 6 (5.2%) | 62 (6.4%) | 0.61 |
| Oral hypoglycemic agents | 67 (24%) | 19 (36%) | 0.06 | 44 (24%) | 33 (28%) | 0.53 | 20 (17%) | 175 (18%) | 0.83 |

AVR=aortic valve replacement, TAVI=transcatheter aortic valve implantation; SAVR=surgical aortic valve replacement; BSA=body surface area; PCI=percutaneous coronary intervention; CABG=coronary artery bypass grafting; STS=society of thoracic surgeons; PROM=predicted risk of mortality; NYHA=New York Heart Association; ACE=angiotensin-converting enzyme; ARB=angiotensin receptor blocker; DOAC=direct oral anticoagulant; H2= histamine type 2 receptor.

**Supplemental Table 5. Patient characteristics in patients with and without dialysis in the SAVR group**

|  | **Without hemodialysis** | **With**  **hemodialysis** | **P value** | **N of patients evaluated** |
| --- | --- | --- | --- | --- |
|  | **(N=475)** | **(N=105)** |  | **(N=580)** |
| **(A) Clinical characteristics** |  |  |  |  |
| Age (years) | 73.7±7.6 | 73.6±7.7 | 0.90 | 580 |
| Age ≥80 years | 90 (19%) | 25 (24%) | 0.26 | 580 |
| Men | 234 (49%) | 70 (67%) | 0.001 | 580 |
| Body mass index (kg/m^2^) | 23.6±3.8 | 21.2±3.1 | <0.001 | 580 |
| Body mass index <22.0 kg/m^2^ | 163 (34%) | 68 (65%) | <0.001 | 580 |
| BSA, m^2^ | 1.6±0.2 | 1.6±0.2 | 0.17 | 580 |
| Systolic blood pressure, mmHg | 132±21 | 136±24 | 0.10 | 451 |
| Diastolic blood pressure, mmHg | 71±13 | 71±15 | 0.68 | 451 |
| Heart rate, beat per minutes | 73±14 | 73±12 | 0.77 | 331 |
| Hypertension | 346 (73%) | 90 (86%) | 0.006 | 580 |
| Current smoking | 36 (7.6%) | 6 (5.7%) | 0.51 | 580 |
| Dyslipidemia | 263 (55%) | 47 (45%) | 0.049 | 580 |
| Diabetes mellitus | 139 (29%) | 38 (36%) | 0.16 | 580 |
| on insulin therapy | 18 (3.8%) | 10 (9.5%) | 0.01 | 580 |
| Prior myocardial infarction | 20 (4.2%) | 4 (3.8%) | 0.85 | 580 |
| Prior PCI | 44 (9.3%) | 19 (18%) | 0.008 | 580 |
| Prior CABG | 2 (0.4%) | 2 (1.9%) | 0.10 | 580 |
| Prior open heart surgery | 10 (2.1%) | 4 (3.8%) | 0.30 | 580 |
| Prior symptomatic stroke | 36 (7.6%) | 17 (16%) | 0.006 | 580 |
| Atrial fibrillation or flutter | 76 (16%) | 28 (27%) | 0.01 | 580 |
| Aortic and/or peripheral vascular disease | 29 (6.1%) | 15 (14%) | 0.004 | 580 |
| eGFR (mL/min/1.73m^2^) | 59.7±18.6 | 6.0±1.9 | <0.001 | 566 |
| eGFR <30 mL/min/1.73m^2^ not on dialysis | 28 (5.9%) | 0 (0.0%) | 0.01 | 580 |
| Serum creatinine | 0.9 (0.7-1.0) | 7.6 (6.1-8.9) | <0.001 | 566 |
| Creatinine level >2 mg/dl or dialysis | 12 ( 2.5%) | 105 (100%) | <0.001 | 580 |
| Anemia | 160 (34%) | 73 (70%) | <0.001 | 580 |
| Liver cirrhosis (Child B or C) | 2 (0.4%) | 1 (1.0%) | 0.49 | 580 |
| Malignancy | 75 (16%) | 23 (22%) | 0.13 | 580 |
| Malignancy currently under treatment | 15 (3.2%) | 7 (6.7%) | 0.09 | 580 |
| Chest wall irradiation | 4 (0.8%) | 1 (1.0%) | 0.91 | 580 |
| Immunosuppressive therapy | 17 (3.6%) | 5 (4.8%) | 0.57 | 580 |
| Chronic lung disease | 144 (30%) | 36 (34%) | 0.43 | 580 |
| Chronic lung disease (moderate or severe) | 15 (3.2%) | 7 (6.7%) | 0.09 | 580 |
| Coronary artery disease | 170 (36%) | 56 (53%) | 0.001 | 580 |
| Clinical Frailty Scale |  |  | <0.001 | 580 |
| 1-3 | 369 (78%) | 54 (51%) |  | 580 |
| 4-6 | 102 (22%) | 49 (47%) |  | 580 |
| 7-9 | 4 (0.8%) | 2 (1.9%) |  | 580 |
| STS PROM, % | 2.6 (1.7-3.6) | 6.5 (4.8-8.6) | <0.001 | 580 |
| EuroSCORE II, % | 2.5 (1.3-4.7) | 4.1 (2.5-7.2) | <0.001 | 580 |
| Logistic EuroSCORE, % | 6.6 (4.4-9.7) | 13.0 (8.3-21.2) | <0.001 | 580 |
| BNP (pg/ml) | 110 (48-293) | 489 (252-1124) | <0.001 | 476 |
| NT-proBNP (pg/ml) | 582 (233-2911) | 25266 (6702-35000) | <0.001 | 85 |
| **(B) Presentation** |  |  |  |  |
| **Etiology** |  |  | <0.001 |  |
| Degenerative | 341 (72%) | 100 (95%) |  | 580 |
| Congenital (unicuspid, bicuspid, or quadricuspid) | 120 (25%) | 5 (4.8%) |  | 580 |
| Rheumatic | 11 (2.3%) | 0 (0.0%) |  | 580 |
| Infective endocarditis | 2 (0.4%) | 0 (0.0%) |  | 580 |
| Other | 1 (0.2%) | 0 (0.0%) |  | 580 |
| **Symptoms** |  |  |  |  |
| Any symptoms probably related to AS | 369 (78%) | 72 (69%) | 0.048 | 580 |
| Chest pain | 81 (22%) | 14 (19%) | 0.64 | 580 |
| Syncope | 35 (9.5%) | 5 (6.9%) | 0.49 | 580 |
| Heart failure | 308 (84%) | 54 (75%) | 0.09 | 580 |
| NYHA class |  |  | 0.04 |  |
| II | 223 (72%) | 33 (61%) |  | 580 |
| III | 62 (20%) | 19 (35%) |  | 580 |
| IV | 23 (7.5%) | 2 (3.7%) |  | 580 |
| **(C) Medications at index echocardiography** |  |  |  |  |
| Antiplatelet therapy |  |  |  |  |
| Aspirin | 99 (21%) | 40 (38%) | <0.001 | 580 |
| Thienopyridine |  |  |  |  |
| Clopidogrel | 28 (5.9%) | 19 (18%) | <0.001 | 580 |
| Prasugrel | 4 (0.8%) | 2 (1.9%) | 0.33 | 580 |
| Ticlopidine | 0 (0.0%) | 0 (0.0%) | - | 580 |
| Statins | 235 (50%) | 39 (37%) | 0.02 | 580 |
| Beta-blockers | 105 (22%) | 37 (35%) | 0.005 | 580 |
| ACE inhibitors/ARB | 231 (49%) | 41 (39%) | 0.08 | 580 |
| Calcium channel blockers | 225 (47%) | 45 (43%) | 0.40 | 580 |
| Warfarin | 26 (5.5%) | 16 (15%) | <0.001 | 580 |
| DOAC | 48 (10%) | 0 (0.0%) | 0.001 | 580 |
| Proton pump inhibitors | 156 (33%) | 57 (54%) | <0.001 | 580 |
| H2 blockers | 26 (5.5%) | 8 ( 7.6%) | 0.40 | 580 |
| Oral hypoglycemic agents | 110 (23%) | 21 (20%) | 0.48 | 580 |

AVR=aortic valve replacement, TAVI=transcatheter aortic valve implantation; SAVR=surgical aortic valve replacement; BSA=body surface area; PCI=percutaneous coronary intervention; CABG=coronary artery bypass grafting; STS=society of thoracic surgeons; PROM=predicted risk of mortality; NYHA=New York Heart Association; ACE=angiotensin-converting enzyme; ARB=angiotensin receptor blocker; DOAC=direct oral anticoagulant; H2= histamine type 2 receptor..

**Supplemental Table 6.** Procedural characteristics of SAVR and TAVI

|  | **SAVR** |
| --- | --- |
|  | **N=580 (%)** |
| Concomitant procedures | 279 (48%) |
| CABG | 161 (28%) |
| Mitral valve surgery | 55 (9.5%) |
| Tricuspid valve surgery | 32 (5.5%) |
| Replacement of ascending aorta | 49 (8.4%) |
| Bentall operation | 4 (0.7%) |
| Annular dilatation | 2 (0.3%) |
| Maze procedure | 43 (7.4%) |
| Bioprosthetic valve | 556 (96%) |
| 19 mm | 109 (20%) |
| 21mm | 222 (40%) |
| 23 mm | 165 (30%) |
| 25 mm | 37 (6.7%) |
| 27 mm | 2 (0.4%) |
| Sutureless valve (Perceval) | 21 (3.6%) |
| S size | 11 (2.0%) |
| M size | 6 (1.1%) |
| L size | 2 (0.4%) |
| XL size | 2 (0.4%) |
| Mechanical valve | 24 (4.1%) |
| ~19 mm | 6 (1.0%) |
| 21 mm | 8 (1.4%) |
| 22 mm | 2 (0.3%) |
| 23 mm | 3 (0.5%) |
| 24 mm | 2 (0.3%) |
| 25 mm | 3 (0.5%) |
|  |  |
|  | **TAVI** |
|  | **N=1134 (%)** |
| General anesthesia | 438 (39%) |
| Local anesthesia with conscious sedation | 696 (61%) |
| Approach site |  |
| Transfemoral approach | 1040 (92%) |
| Transapical approach | 28 (2.5%) |
| Transilliac approach | 1 (0.1%) |
| Transsubclavian approach | 7 (0.6%) |
| Transbrachiocephalic approach | 1 (0.1%) |
| Direct aorta approach | 57 (5.0%) |
| PCI before TAVI |  |
| Separate session from TAVI | 68 (6.0%) |
| Same session with TAVI | 10 (0.9%) |
| IABP support | 10 (0.9%) |
| PCPS support | 18 (1.6%) |
| Valve type |  |
| SAPIEN 3 | 788 (70%) |
| 20 mm | 42 (5.3%) |
| 23 mm | 379 (48%) |
| 26 mm | 289 (37%) |
| 29 mm | 78 (9.9%) |
| Evolut R | 96 (8.5%) |
| 23 mm | 15 (16%) |
| 26 mm | 52 (54%) |
| 29 mm | 25 (26%) |
| 34 mm | 4 (4.2%) |
| Evolut PRO/PRO+ | 248 (22%) |
| 23 mm | 29 (12%) |
| 26 mm | 143 (58%) |
| 29 mm | 73 (30%) |
| 34 mm | 2 (0.8%) |
| No valve* | 2 (0.2%) |

*Procedural failure because of access route complication before implanting valve.

CABG＝coronary artery bypass grafting; IABP= intra-aortic balloon pumping; PCPS= percutaneous cardiopulmonary support.

**Supplemental Table 7.** In-hospital outcomes in patients without dialysis

|  | **SAVR group** | **TAVI group** |
| --- | --- | --- |
|  | **(N=475)** | **(N=1132)** |
| All-cause death | 4 (0.8%) | 7 (0.6%) |
| Stroke | 7 (1.5%) | 32 (2.8%) |
| Disabling stroke^†^ | 2 (0.4%) | 21 (1.9%) |
| Major Bleeding^‡^ | 322 (68%) | 221 (20%) |
| Newly diagnosed AF | 122 (26%) | 52 (4.6%) |
| Pacemaker implantation | 12 (2.5%) | 92 (8.1%) |
| Major vascular complication^§^ | 12 (2.5%) | 26 (2.3%) |
| ICU stay after procedure (days) | 3 (2-4) | 1 (1-2) |
| Hospital stay after procedure (days) | 15 (12-21) | 8 (7-12) |

ICU=intensive care unit; AF=atrial fibrillation.

**Supplemental Table 8.** In-hospital outcomes in patients with dialysis

|  | **SAVR group** | **TAVI group** |
| --- | --- | --- |
|  | **(N=105)** | **(N=2)** |
| All-cause death | 9 (8.6%) | 0 (0.0%) |
| Stroke | 5 (4.8%) | 0 (0.0%) |
| Disabling stroke | 3 (2.9%) | 0 (0.0%) |
| Major Bleeding | 94 (90%) | 1 (50%) |
| Newly diagnosed AF | 31 (30%) | 0 (0.0%) |
| Pacemaker implantation | 2 (1.9%) | 0 (0.0%) |
| Major vascular complication | 8 (7.6%) | 0 (0.0%) |
| ICU stay after procedure (days) | 4 (3-5) | 10 (6.5-13.5) |
| Hospital stay after procedure (days) | 22 (16-34) | 20.5 (20.25-20.75) |

ICU=intensive care unit; AF=atrial fibrillation.

**Supplemental Table 9.** In-hospital outcomes in patients who underwent SAVR with/without concomitant procedure

|  | **With concomitant procedure** | **Without concomitant procedure** |
| --- | --- | --- |
|  | **(N=279)** | **(N=301)** |
| All-cause death | 12 (4.3%) | 1 (0.3%) |
| Stroke | 7 (2.5%) | 5 (1.7%) |
| Disabling stroke | 2 (0.7%) | 3 (1.0%) |
| Major Bleeding | 241 (86%) | 175 (58%) |
| Newly diagnosed AF | 81 (29%) | 72 (24%) |
| Pacemaker implantation | 9 (3.2%) | 5 (1.7%) |
| Major vascular complication | 11 (3.9%) | 9 (3.0%) |
| ICU stay after procedure (days) | 3 (2-5) | 3 (2-3) |
| Hospital stay after procedure (days) | 20 (14-28) | 15 (12-19) |

ICU=intensive care unit; AF=atrial fibrillation.

**Supplemental Table 10.** In-hospital outcomes in patients with bicuspid valve who underwent SAVR and TAVI

|  | **SAVR** | **TAVI** |
| --- | --- | --- |
|  | **(N=121)** | **(N=34)** |
| All-cause death | 1 (0.8%) | 1 (2.9%) |
| Stroke | 1 (0.8%) | 2 (5.9%) |
| Disabling stroke | 0 (0.0%) | 2 (5.9%) |
| Major Bleeding | 72 (60%) | 5 (15%) |
| Newly diagnosed AF | 32 (26%) | 4 (12%) |
| Pacemaker implantation | 1 (0.8%) | 1 (2.9%) |
| Major vascular complication | 5 (4.1%) | 1 (2.9%) |
| ICU stay after procedure (days) | 2 (2-3) | 1 (1-2) |
| Hospital stay after procedure (days) | 7 (5-10) | 14 (11-17) |

ICU=intensive care unit; AF=atrial fibrillation.

**Supplemental Table 11. In-hospital mortality according to STS score categories**

| **STS-PROM score** | **SAVR group** | **TAVI group** |
| --- | --- | --- |
|  | **(N=580)** | **(N=1134)** |
| **High risk*** | 6/51 (11.8%) | 2/157 (1.2%) |
| **Intermediate risk** | 4/132 (3.0%) | 4/538 (0.7%) |
| **Low risk** | 3/397 (0.8%) | 1/439 (0.2%) |

STS-PROM = Society of Thoracic Surgeons Predicted Risk of Mortality; TAVI=transcatheter aortic valve implantation; SAVR=surgical aortic valve replacement.

* High risk was defined as STS-PROM score < 4% , intermediate risk was defined as 4% ≤ STS-PROM score < 8%, and low risk was defined as 8% ≤ STS-PROM score.

**Supplemental Table 12. In-hospital mortality in patients with CAD**

|  | **SAVR group** | **TAVI group** |
| --- | --- | --- |
|  | **(N=226)** | **(N=448)** |
| All-cause death | 10 (4.4%) | 4 (0.4%) |

CAD= Coronary artery disease. Coronary artery disease was defined to be present when meeting at least one of the following criteria: a history of PCI or CABG, a history of myocardial infarction, presentation as myocardial infarction at baseline, or angiographically confirmed coronary artery disease at baseline.

**Supplemental Table 13. In-hospital mortality in patients with any valvular disease**

|  | **SAVR group** | **TAVI group** |
| --- | --- | --- |
|  | **(N=161)** | **(N=258)** |
| All-cause death | 9 (5.6%) | 1 (0.4%) |

Any combined valvular disease was defined condition in which at least one of AR, MS, MR, and TR is moderate or severe.

**Supplemental Figure 1.** **Distribution of STS score in the SAVR and TAVI groups**

Reference:

1. Kappetein AP, Head SJ, Généreux P, et al. Updated standardized endpoint definitions for transcatheter aortic valve implantation: The valve academic research consortium-2 consensus document (varc-2). *Eur J Cardio-thoracic Surg*. 2012;42.

2. Thygesen K, Alpert JS, White HD, et al. Universal definition of myocardial infarction. *Circulation*. 2007;116:2634–2653.

3. Généreux P, Piazza N, Alu MC, et al. Valve Academic Research Consortium 3: updated endpoint definitions for aortic valve clinical research. *Eur Heart J*. 2021;42:1825–1857.

4. Mehran R, Rao S V, Bhatt DL, et al. Standardized bleeding definitions for cardiovascular clinical trials: a consensus report from the Bleeding Academic Research Consortium. *Circulation*. 2011;123:2736–2747.
